# Estimating migratory connectivity of birds when re-encounter probabilities are heterogeneous

**DOI:** 10.1002/ece3.1059

**Published:** 2014-04-08

**Authors:** Emily B Cohen, Jeffrey A Hostetler, J Andrew Royle, Peter P Marra

**Affiliations:** 1 Migratory Bird Center, Smithsonian Conservation Biology Institute, National Zoological ParkWashington, District of Columbia; 2 U.S. Geological Survey, Patuxent Wildlife Research CenterLaurel, Maryland

**Keywords:** Bird Banding Laboratory, migratory connectivity, multistate model, Nearctic-Neotropical Migrant, re-encounter probability

## Abstract

Understanding the biology and conducting effective conservation of migratory species requires an understanding of migratory connectivity – the geographic linkages of populations between stages of the annual cycle. Unfortunately, for most species, we are lacking such information. The North American Bird Banding Laboratory (BBL) houses an extensive database of marking, recaptures and recoveries, and such data could provide migratory connectivity information for many species. To date, however, few species have been analyzed for migratory connectivity largely because heterogeneous re-encounter probabilities make interpretation problematic. We accounted for regional variation in re-encounter probabilities by borrowing information across species and by using effort covariates on recapture and recovery probabilities in a multistate capture–recapture and recovery model. The effort covariates were derived from recaptures and recoveries of species within the same regions. We estimated the migratory connectivity for three tern species breeding in North America and over-wintering in the tropics, common (*Sterna hirundo*), roseate (*Sterna dougallii*), and Caspian terns (*Hydroprogne caspia*). For western breeding terns, model-derived estimates of migratory connectivity differed considerably from those derived directly from the proportions of re-encounters. Conversely, for eastern breeding terns, estimates were merely refined by the inclusion of re-encounter probabilities. In general, eastern breeding terns were strongly connected to eastern South America, and western breeding terns were strongly linked to the more western parts of the nonbreeding range under both models. Through simulation, we found this approach is likely useful for many species in the BBL database, although precision improved with higher re-encounter probabilities and stronger migratory connectivity. We describe an approach to deal with the inherent biases in BBL banding and re-encounter data to demonstrate that this large dataset is a valuable source of information about the migratory connectivity of the birds of North America.

## Introduction

Understanding the biology and conducting effective conservation of migratory species requires knowledge of migratory connectivity, the geographic linkage of individuals, or populations between phases of the annual cycle (Marra et al. 2010). Quantifying the degree to which individuals from a breeding population move to the same nonbreeding region is necessary for understanding how events during one phase of the annual cycle influence subsequent phases (Marra et al. 2005; Runge and Marra 2005; Webster and Marra 2005). For example, habitat or precipitation experienced during the nonbreeding periods can influence an individual's survival to breeding (Wilson et al. 2011), arrival timing (Tøttrup et al. 2008, 2012; McKellar et al. 2012), or reproductive success (Norris et al. 2004). Therefore, knowledge of migratory connectivity is essential for understanding population regulation, projecting the impacts of future threats, and solving difficult environmental issues such as the spread of infectious disease (Webster and Marra 2005; Sheehy et al. 2010; Henkel et al. 2012).

Despite its importance, relatively little is known about the migratory connectivity of North American birds (Veen 2013). At present, we know the basic breeding and stationary nonbreeding ranges of most North American breeding species, but we do not know how populations are linked between these areas, including some of the most studied species in North America. Migratory connectivity has been quantified for a few North American species, particularly game species that migrate within North America (e.g., Diefenbach et al. 1988; Hestbeck et al. 1991). However, these studies typically provide estimates for only a few sites within the ranges of the species and have generally not accounted for the spatial variability in finding and reporting of re-encountered birds.

New tracking devices are providing information about migratory connectivity (Hobson 2003; Wikelski et al. 2007; Bridge et al. 2011; Douglas et al. 2012). However, current tracking methodologies have limitations. Satellite tracking devices remain too large for many species, and while they provide considerable data for each tagged individual, they are expensive to deploy on a large scale (Bridge et al. 2011). Geolocation dataloggers have limited precision and require that the animal be recaptured to retrieve data (Fudickar et al. 2011; Lisovski et al. 2012). Genetic markers, stable isotope signatures, morphology, and band recoveries are increasingly integrated to estimate migratory connectivity (Rundel et al. 2013; Rushing et al. 2014). These estimates from multiple data sources are more precise than estimates derived from single sources, but precision is still limited and modeling frameworks are not straightforward (Rundel et al. 2013).

On the other hand, banding and re-encounter data are the most spatially accurate source of information on migratory connectivity for many species. The North American Bird Banding Laboratory (BBL) may be the largest inventory of tagged vertebrates in the western hemisphere (http://www.pwrc.usgs.gov/bbl). It houses over 70 million banding and 4.5 million re-encounter records (live resightings or recaptures and dead recoveries) and is the only long-term dataset available for most Nearctic breeding bird species. The BBL database records include when, where, and how individuals were banded (from 1955 to present) and re-encountered (from 1914 to present). The BBL database maintains the original banding records necessary to build individual capture histories, data not available within the European EURING databank (http://www.euring.org, Korner-Nievergelt et al. 2012).

Given its availability as a source of information about migratory connectivity, banding data have too often been dismissed and underutilized (Korner-Nievergelt et al. 2010a, 2012). Most efforts to interpret long-distance geographic linkages from banding and re-encounter records have mapped the raw data (e.g., Brewer 2000; Bønløkke et al. 2006; Sharrock 2010). However, these maps cannot be directly equated with population distributions because they do not account for the geographic variation in banding effort and re-encounter probabilities (Nichols 1996; Kendall et al. 2006; Korner-Nievergelt et al. 2010a). Spatial heterogeneity in finding and reporting of banded birds has long been documented (Nichols et al. 1995; Royle and Dubovsky 2001). For example, there is a high concentration of common tern (*Sterna hirundo*) band recoveries in Guyana where they have been frequently trapped for food (Hays et al. 1997). However, common terns may be equally abundant in other parts of their nonbreeding range where trapping does not occur. Thus, interpretation of re-encounter locations requires accounting for the variation in the data due to the unknown observer and reporting distribution (Korner-Nievergelt et al. 2010a).

There are well-developed statistical techniques for estimating movement probabilities from capture–recapture and recovery data that deal with the influence of the observer process by incorporating parameters for re-encounter probabilities (Brownie et al. 1993; Gimenez et al. 2007; Gauthier and Lebreton 2008). However, the small proportion of birds banded in breeding areas that are re-encountered in nonbreeding areas often limit the number of parameters that can be estimated with these data (Korner-Nievergelt et al. 2010a,b). Therefore, statistical methods to overcome the issue of heterogeneous re-encounter probabilities in the face of small sample sizes are necessary to make large-scale banding and re-encounter data an available source of information about migratory connectivity.

In this paper, we present a novel and broadly applicable approach for deriving migratory connectivity estimates from banding and re-encounter data that accounts for heterogeneous re-encounter probabilities with limited data. We illustrate our approach with three species of tern that breed in North America, common tern, roseate tern (*Sterna dougallii*), and Caspian tern (*Hydroprogne caspia*). Finally, we use simulated data to evaluate the applicability of the method to species that vary in the number of individuals banded and their re-encounter probabilities.

## Methods

### Model development

The objective of this study was to estimate migratory connectivity from breeding to stationary nonbreeding regions using banding capture, recapture, and recovery data. We used Burnham's live-recapture dead-recovery modeling framework (Burnham 1993; Williams et al. 2002) which has four parameters, survival probability (*φ*), recapture probability (*p*), recovery probability (*r*), and migration (transition) probability (*π*, Table 1). The model incorporated all individuals banded in breeding regions and any re-encounters in stationary nonbreeding regions. We analyzed data in the R (R Development Core Team 2012) package RMark (Laake and Rexstad 2008) which is an interface for program MARK (White and Burnham 1999).

**Table 1:** Description of parameters used in the multistate live and dead re-encounter models

Name	Description
*π*_*ij*_	Probability of a bird breeding in site *i* spends the nonbreeding season in site *j*
*r*_*j*_	Probability that a bird dead in nonbreeding region *j* is found and reported
*p*_*j*_	Probability that a bird alive in nonbreeding region *j* is seen or captured and reported
*φ*	Annual apparent survival probability
*R*_*j*_	Site-specific effort covariate of dead re-encounter and reporting probability derived from the number of individuals of many species recovered in region *j* during nonbreeding
*P*_*j*_	Site-specific effort covariate of live re-encounter and reporting probability derived from the number of individuals of many species recovered in region *j* during nonbreeding

The state transition occurred once, from one breeding to one nonbreeding region. We model the encounter histories of birds captured and released and either recaptured, resighted, recovered, or never re-encountered again. For example, the capture histories below are in the live dead (LD) format, consisting of three pairs of columns representing three capture occasions followed by their probability structure (Burnham 1993; White and Burnham 1999):


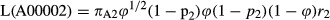



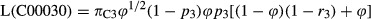


The first occasion is for half of a year, from summer breeding to winter nonbreeding. Consequently, survival was calculated for half of the year for the first occasion, breeding to nonbreeding season, and was annual thereafter, nonbreeding to nonbreeding season. During the first occasion, birds were banded in breeding areas A and C, respectively. The first dead occasion is always zero because recovery within the breeding season was not incorporated in the model. In both of the capture histories, birds were not re-encountered live or dead during the first winter after banding. The bird in the first example banded in breeding region A was found dead in nonbreeding region 2 during the second winter. The bird in the second example banded in breeding region C was recaptured or resighted in nonbreeding region 3 during the second winter. We followed the Burnham model formulation which makes it possible for a recapture and recovery to occur in the same year. For simplicity, the model assumes that recaptures happen during discrete capture occasions, but recoveries are not restricted to those occasions (Burnham 1993; Williams et al. 2002). This is generally not the case for large-scale banding data, where both recaptures and recoveries happen throughout the nonbreeding season. However, estimates of migratory connectivity were robust to this violation in preliminary simulations (J. Hostetler, unpublished analysis). Therefore, in the first capture history example, the bird survives one and one half years (*φ*^1/2^ *φ*(1-*φ*)) because recovery is assumed to have occurred between years. In the second example, the bird either dies after the last capture but is not recovered ((1−*φ*)(1−*r*_3_)) or survives (*φ*).

We increased parameter identifiability by estimating parameters associated with the sampling process, recapture (*p*_j_), and recovery (*r*_j_) probability, as the same among similar sized species that occupy overlapping nonbreeding habitats and regions (Thorup and Conn 2009). When one of these species did not occur in one or more of the nonbreeding regions, we fixed migratory connectivity parameters to zero (White et al. 2006), providing known estimates for those *π*_*ij*_ (Thorup and Conn 2009; Korner-Nievergelt et al. 2010b).

BBL re-encounter data for nongame species are not systematically acquired; rather they are the long-term result of a combination of information from local-scale research projects, large-scale monitoring programs, and public reporting of band recoveries. Therefore, we estimated both recapture and recovery probabilities because the processes behind the recapture and recovery probabilities are likely to differ (Kendall et al. 2006). We took advantage of the large BBL database to build “effort” covariates for nonbreeding regions. The effort covariates borrowed data across many species to estimate the likelihood that a banded bird would be re-encountered in a nonbreeding region during the over-wintering period. We extracted records from the BBL database that occurred during the over-wintering months within the nonbreeding ranges of the species modeled. Of those records, we included species that were similar in size and habitat affiliation to the modeled species. The migratory connectivity of these species was not modeled due to low species-specific re-encounter numbers. The region-specific live (*P*_*j*_) and dead (*R*_*j*_) effort covariates (Table 1) were calculated as the total number of re-encounters that fit our criteria in each region divided by the area of that region. They were incorporated into the re-encounter probabilities (*p*_j_ and *r*_j_, respectively) with a logit link (Data S1).

Application of this model requires assumptions important to consider when selecting species, and defining the spatial and temporal scales of interest. First, multistate mark-recapture models assume individuals within a state and capture occasion all have the same probability of detection, survival, and transition to the next occasion. The models also assume that the fate of each bird is correctly assigned, with no tag loss, no influence from bands, and independence with the fate in the next occasion with respect to detection and survival. We also assumed that survival and detection during the nonbreeding period were independent of the breeding region. Re-encounter probabilities were assumed to be constant among species within over-wintering regions. Therefore, we chose species that overlapped in their nonbreeding ranges and were similar in size and habitat affiliations. We modeled species similar in size and habitat affiliations to minimize variability between species in finding and reporting of recoveries.

The temporal scale selected was assumed to incorporate the full time periods when birds occurred in stationary regions, breeding and nonbreeding. We incorporated all available banding and re-encounter data, but reduced the number of parameters in our model by constraining them to be equal over years. Thus, we assumed that there was no temporal variability in migratory connectivity, survival, or re-encounter probabilities from 1955 through 2011. While re-encounter records are available as early as 1914, computerized banding data are available and records were included for 1955 through 2011. Shorter time periods may be more appropriate for some BBL species (Visser et al. 2009).

The spatial scales of nonbreeding regions were chosen to incorporate the full nonbreeding regions of species and individuals. We assumed that the entire nonbreeding range was incorporated to ensure that migratory connectivity parameters sum to one. We also assumed that individuals in this model used only one breeding and one nonbreeding region during their lifetime. To avoid violation of this assumption, we assigned large-scale breeding and nonbreeding regions, and evaluated these designations with BBL data. No movement of any individuals of the study species was observed between breeding regions or between nonbreeding regions.

### Example using three tern species

Common, roseate, and Caspian terns are long-distance migrants that breed in North America and have largely overlapping nonbreeding ranges (Gochfeld et al. 1998; Cuthbert and Wires 1999; Nisbet 2002). We estimated migratory connectivity for these three species from breeding areas in the Northeast coast (“Eastern”), around the Great Lakes and along the St. Lawrence River (“Central”), and the interior West and Pacific coast (“Western”) of North America to the coasts of four nonbreeding regions, (1) the Southern U.S. along the Gulf Coast and Florida and the Caribbean (GULF.CARIB), (2) eastern South America (ESAM), (3) Mexico and Central America (CAM), and (4) western South America (WSAM).

Common terns have been extensively banded in their Atlantic Coast breeding colonies but less so in Central and Western parts of their North American breeding range (Nisbet 2002). Roseate terns are concentrated in a few major breeding colonies along the North Atlantic Coast of North America (Gochfeld et al. 1998). Less is known about the nonbreeding distribution of roseate terns, but they occur together with common terns in South America and have also been detected in the Caribbean (Nisbet 1984; Gochfeld et al. 1998; Hays et al. 1999). Caspian terns have a broad breeding distribution on coastlines, and inland lakes and rivers in North America but are locally uncommon in most parts of their range (Cuthbert and Wires 1999; Wires and Cuthbert 2000; Morris et al. 2010).

We incorporated all individuals that were banded during the breeding region and season, and re-encounter that occurred in the nonbreeding range and season. These species are primarily on breeding grounds during May through August. Less is known about arrival and departure timing in nonbreeding areas, but they have been consistently observed from November through February in their nonbreeding ranges (Nisbet 1984; Cuthbert and Wires 1999; Hays et al. 1999; Nisbet et al. 2011). We included only re-encounters within 10 years of banding, encompassing the life span of most individuals for all three species (Cuthbert and Wires 1999; Hays et al. 1999). We excluded re-encounters where the status of the bird or band was unknown after the re-encounter. Re-encounter records were categorized as (1) resighted or recaptured and released alive, (2) found dead or killed upon capture, or (3) removed from the marked population because the band was removed or the bird was held in captivity (Table 2).

**Table 2:** The number of terns banded during breeding and the percentage of those re-encountered during non-breeding. Of those re-encountered, the status of the bird was live, dead, or unbanded after the re-encounter. Re-encounters of the tern species and the species in the effort covariate were obtained by non-scientists (shot, found dead, trapped) or scientists (recapture or resight). The effort covariate includes re-encounters of coastal species in the non-breeding regions during the winter

	Banded	Re-encounters					
		
			Status			How obtained	
				
	Total	Total, %	Dead, %	Live, %	Unbanded, %	Non-scientific, %	Scientific, %
Common tern	1,059,357	0.09	43.3	42.5	14.2	76.1	23.9
Roseate tern	104,204	0.21	19.6	72.1	8.2	60.7	39.3
Caspian tern	75,580	0.16	83.7	9.8	6.5	94.3	5.7
Effort covariate			49.7	44.6	5.7	64.1	35.9

We built effort covariates of the number of individuals of similar species re-encountered within the nonbreeding regions during over-wintering months. Common, roseate, and Caspian terns are known to occur on beaches and other habitats with other species in their nonbreeding ranges (Blokpoel et al. 1984; Hays et al. 1999; Olmos 2002; Bugoni and Vooren 2005). Thus, we included re-encounters of species represented in orders also commonly found in coastal habitats (Charadriiformes, Gaviiformes, Podicipediformes, Gruiformes, Pelecaniformes, Suliformes, Ciconiiformes, and Procellariiformes). We excluded Anseriiformes because it includes many game species and we expected the re-encounter probabilities from hunted species to be different from species that are not commonly hunted, including the three tern species. We compare re-encounter probability estimates and standard errors with and without the effort covariate.

### Simulation to assess bias and precision

Simulated data were used to evaluate the broader applicability of this model. We simulated data that reflect available sample sizes for species in the North American BBL dataset. We have information about the number of re-encounters of 355 of the approximately 400 species of migratory North American breeding birds in the BBL database. Of those, 77 species had >100 breeding to nonbreeding re-encounter records. The number of individuals re-encountered is a function of the number of birds banded during breeding and re-encounter probabilities during the nonbreeding period. The degree of migratory connectivity, the extent to which birds from the same breeding area migrate to the same nonbreeding area (Marra et al. 2010), may also influence the number of re-encounters. We assessed the magnitude and direction of bias when the strength of re-encounter probabilities or migratory connectivity varied. Where data were poor, very low re-encounter probability and weak connectivity, we expected greater bias. Therefore, the 27 scenarios varied in the number of birds banded (40, 400, and 500 thousand), re-encounter probability (moderate, low, very low), and the strength of migratory connectivity (weak, moderate, and strong).

We simulated migratory connectivity of one species from four breeding regions to four nonbreeding areas. We made the assumption that the number of individuals banded was the same in each breeding area. Re-encounter probabilities were the same in three of the four nonbreeding regions. Re-encounter probability was higher in the fourth nonbreeding region. The numbers of birds migrating to each nonbreeding region varied with the strength of migratory connectivity to that region. We modeled both recapture and recovery with the same effort covariate. There were 100 replicates of 27 scenarios for which we calculated the mean number of individuals re-encountered, coverage (proportion of estimates with confidence intervals that overlap the true value), root-mean-squared error (RMSE, a measure of the difference between actual and estimated values), and bias (difference between the mean of the estimates and the true value).

## Results

**Figure 1:**
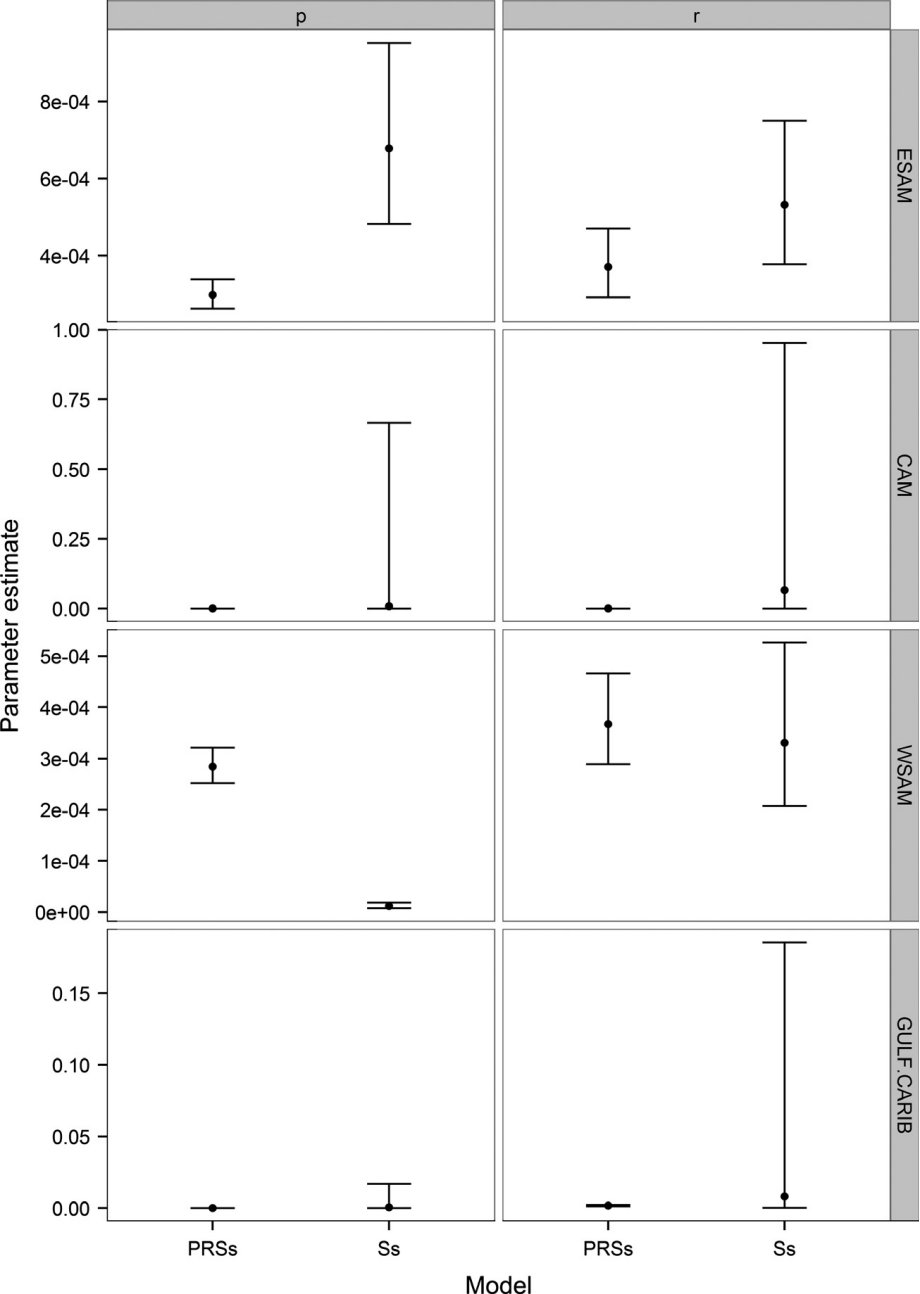
Re-encounter probability estimates for common (*Sterna hirundo*), roseate (*Sterna dougallii*), and Caspian terns (*Hydroprogne caspia*) in four nonbreeding range regions (see Table 3). Estimates are from two models, with (PRSs) and without (Ss) an effort covariate on live, p, and dead, r, re-encounter probabilities (±95% CI). Parameters are assumed to be the same all three species in both models.

We compared model results with and without the effort covariate. Recapture and recovery probability error was smaller in the model with the effort covariate, in all but one case (Fig. 1). Therefore, we present migratory connectivity estimates only from the model with the effort covariate but provide the code to run both models (Data S1). The ratio between the point estimates of recapture and recovery was small in all but three cases (range of values 0.90–4.65). However, recapture and recovery probabilities in Central America were higher in the model that included effort (39.17 and 151.03 times higher, respectively) and recapture in the Gulf and Caribbean was moderately higher (13.26 times higher). The proportion of banded birds that were re-encountered varied among the species, but were fairly consistent within species (Table S1).

**Figure 2:**
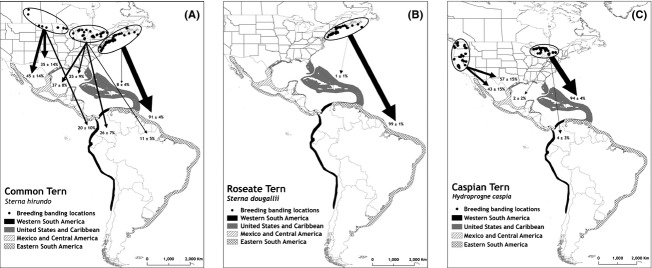
Migratory connectivity estimates for common (A, *Sterna hirundo*), roseate (B, *Sterna dougallii*), and Caspian terns (C, *Hydroprogne caspia*) breeding in North America. The width of the line is proportional to the strength of the connectivity. Estimates ± SE are shown. Less than 1% of Eastern breeding common terns also migrated to Mexico and Central America and western South America.

When re-encounter probability was accounted for, western breeding terns had greater connectivity to the southern US (Table 3). Migratory connectivity estimates for eastern and central breeding terns were moderately different when re-encounter probability was accounted for (Table 3). Eastern breeding terns were strongly connected to eastern South America, and Western breeding birds were strongly tied to the western part of their nonbreeding range (Table 3, Fig. 2). In fact, we found no linkage between western breeding birds and eastern South America. Central breeding common terns were more broadly connected to eastern and western nonbreeding regions (Fig. 2). The least precise estimates of migratory connectivity were for western breeding common terns where fewer individuals were banded. The percentage of birds banded within the breeding areas that were ever re-encountered was low for all three species (Table 2), but similar between breeding regions for each species (Table S1). Annual apparent survival was higher for roseate (0.79 ± 0.02) and similar for common (0.63 ± 0.01) and Caspian terns (0.67 ± 0.03).

**Table 3:** Migratory connectivity estimates (SE) from the proportion of re-encounters in each nonbreeding region, and the multistate live and dead re-encounter model with the effort covariate. Breeding areas are in North America on the Northeast coast (Eastern), around the Great Lakes and along the St. Lawrence River (Central), and the interior West and Pacific coast (Western). Nonbreeding regions are along the coasts of the Southern U.S. and the Caribbean (GULF.CARIB), eastern South America (ESAM), Mexico and Central America (CAM), and western South America (WSAM)

	Common tern			Roseate tern	Caspian tern	
			
	Breeding					
	
Nonbreeding	Eastern	Central	Western	Eastern	Central	Western
	Proportion of re-encounters					
ESAM	0.95 (0.01)	0.12 (0.03)	0 (0)	0.98 (0.01)	0.03 (0.02)	0 (0)
CAM	0.01 (0.003)	0.38 (0.05)	0.55 (0.09)		0.10 (0.03)	1.00 (0)
WSAM	0.01 (0.004)	0.30 (0.05)	0.21 (0.08)	0.01 (0.01)	0.15 (0.04)	0 (0)
GULF.CARIB	0.03 (0.006)	0.21 (0.04)	0.24 (0.08)	0.01 (0.01)	0.72 (0.05)	0 (0)

	Model estimates					
ESAM	0.91 (0.04)	0.11 (0.05)	0 (0)	0.99 (0.01)	0 (0)	0 (0)
CAM	0.01 (0.01)	0.37 (0.08)	0.45 (0.14)		0.02 (0.02)	0.43 (0.15)
WSAM	0.01 (0.003)	0.26 (0.07)	0.20 (0.10)	0.01 (0.01)	0.04 (0.03)	0 (0)
GULF.CARIB	0.08 (0.04)	0.25 (0.09)	0.35 (0.14)	0 (0)	0.94 (0.04)	0.57 (0.15)

**Figure 3:**
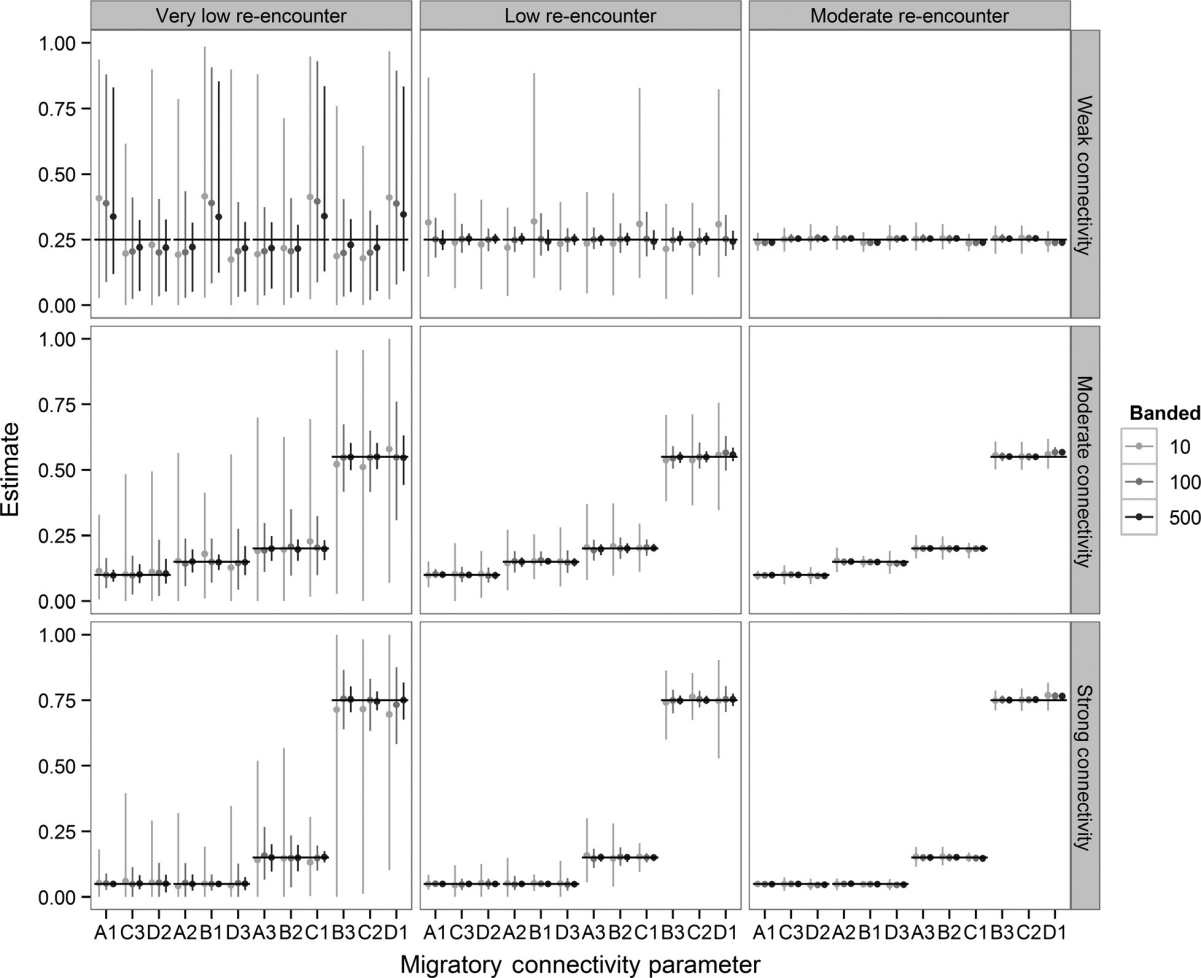
Migratory connectivity estimates from simulated data. Birds from each of four breeding areas (A–D) migrate to each of four stationary nonbreeding areas (1–4). Mean estimates (maximum and minimum values) are from 100 replicates of 27 scenarios. Scenarios varied in the number of birds banded in each of four breeding areas (10, 100, and 500 thousand), the strength of migratory connectivity, and re-encounter probabilities. The strength of migratory connectivity is a 4 × 4 matrix with 16 values (weak all *π*_*ij*_ = 0.25, moderate *π*_*ij*_ = 0.10, 0.15, 0.20, 0.55, and strong *π*_*ij*_ = 0.05, 0.05, 0.15, 0.75). One migratory connectivity parameter from each breeding area (A–D) is calculated as one minus the sum of the other three. So, 12 of the 16 migratory connectivity parameters are estimated and presented here. In each scenario, re-encounter probability is higher in one nonbreeding area (1 is very low: 0.0015, low: 0.01, moderate: 0.08) and the same in the other nonbreeding areas (2–4 are very low: 0.0002, low: 0.002, moderate: 0.01). The solid lines indicate the true values.

The simulations demonstrate that the accuracy and precision of migratory connectivity estimates generally increased with re-encounter probabilities and number of birds banded (Fig. 3, Table S2). Coverage was 85–100% across scenarios except when re-encounter probabilities and number of birds banded were lowest and when re-encounter probabilities and number of birds banded were highest (Table S2). The difference between actual and observed values (RMSE) was highest when re-encounter probabilities and number of birds banded were low (Table S2). However, when data were poor, with very low re-encounter probability and weak migratory connectivity, the model overestimated the effect of the effort covariate. Migratory connectivity estimates to nonbreeding areas with higher re-encounter probabilities were biased high while estimates to areas with lower re-encounter probabilities were biased low. There was little evidence of bias for all other cases (Table S2).

## Discussion

Most of the breeding birds of North America are represented in what is likely the largest database of tagged vertebrates in the western hemisphere. We explore the use of these data to derive estimates of migratory connectivity from models that account for re-encounter probability. Using simulations, we show that the model is likely applicable for BBL species with >40,000 birds banded. Although, precision of estimates improve as the number of birds banded, re-encounter probability, and the strength of migratory connectivity increases. There was also little evidence of bias except for when migratory connectivity was weak and re-encounter probability was very low. Re-encounter probabilities are likely to vary with habitat affiliations and body size but, in general, they are likely to be relatively low in tropical nonbreeding regions. Low re-encounter probabilities will, therefore, likely limit the accuracy and precision of migratory connectivity estimates for many species in the BBL database.

For western breeding terns, model-derived estimates of migratory connectivity differed considerably from those derived directly from the proportions of re-encounters. Conversely, for eastern breeding terns, estimates were merely refined by the inclusion of re-encounter probabilities. The proportion of banded birds that were re-encountered (in any nonbreeding region) was fairly consistent between breeding regions within species. However, the number of birds banded in breeding regions varied considerably. Fewer birds were banded in the west and, therefore, fewer were also re-encountered. If this concentration of banding effort is common for other species, then precision of estimates are likely to reflect these geographic biases. However, we also found that most breeding regions were strongly connected to one or more nonbreeding region. If this pattern of strong connectivity is common, it should increase our ability to estimate migratory connectivity from these data (Korner-Nievergelt et al. 2012).

Migratory connectivity information from additional data sources, such as tracking devices, will be useful for verifying results of these models. Five Eastern common terns tracked using geolocators spent the nonbreeding season along the eastern coast of South America with some individuals using more than one area during the same year but not moving between the regions we designated (Nisbet et al. 2011). Further, re-encounter data prior to 1955 were not included in the model because the numbers of birds banded were unknown. However, it is possible to estimate the numbers of birds banded from breeding to breeding re-encounters (Korner-Nievergelt et al. 2012), making it possible to compare estimates from these data. We divided the nonbreeding ranges into a few large regions in an effort to meet the assumptions that all potential nonbreeding regions were incorporated and that individuals did not change breeding or nonbreeding regions during their lifetime. We did not detect any terns changing nonbreeding regions either within or between years. Fidelity to natal colony was high for Eastern breeding common and roseate terns (Spendelow et al. 1995; Nisbet and Spendelow 1999). Western Caspian terns moved between breeding colonies, but remained within the breeding region (Wires and Cuthbert 2000).

The proportion of banded birds of each tern species that were re-encountered varied among the species despite their overlapping habitat associations and similarity in size. Re-encounter probabilities are likely to differ somewhat between species due to differences in such things as body size, habitat associations, behavior, and research effort. It is difficult to assess the extent to which variability among species is due to species-specific differences in re-encounter probabilities or the difference in the proportional linkages of species to nonbreeding regions that differ in re-encounter probabilities. Future work could compare estimates when re-encounter probabilities are modeled as proportional, instead of equal, between the three species (Korner-Nievergelt and Hofer 2009). The assumption that there was no difference in re-encounter probabilities between breeding regions was also difficult to evaluate because re-encounter probabilities differ between the nonbreeding regions and the species migrate to the nonbreeding regions in different proportions. We also could not assess whether re-encounter probabilities were equal within nonbreeding regions with our dataset. Korner-Nievergelt et al. (2012) found that violation of this assumption did not strongly bias parameter estimates for one species, but this result may not be generalizable to other species.

Currently, we know year-round ranges for many species but not how migratory populations are connected throughout the annual cycle. This missing information is needed to understand the factors that regulate migratory populations and to forecast how climate change will affect the biology of migratory species. With over 70 million birds banded and over 1.2 million new records added every year, the methods presented here make the BBL database an important untapped resource for building a broader understanding of many of the migratory birds of North America. These data can provide baseline estimates of migratory connectivity that can be verified or improved with additional information from band sighting databases (e.g., http://www.mybandedtern.org, http://www.bandedbirds.org, Kendall et al. 2006; Gratto-Trevor et al. 2012), stable isotopes, genetics, and tracking devices (Boulet et al. 2006; Ryder et al. 2011; Fraser et al. 2012; Macdonald et al. 2012). Large-scale banding and re-encounter data are available for many species, and it is possible to deal with the biases in these data to estimate migratory connectivity.

## Supporting Information

**Table S1.** The number of birds banded in each breeding region and re-encountered in each nonbreeding region, and the percent of birds banded that were encountered.

**Table S2.** The model precision and bias from 100 replicates of simulated data.

**Data S1.** R Code for MSLiveDead Mark models and Simulation analyses in this paper.

## References

[b1] Blokpoel H, Morris RD, Tessier GD (1984). Field investigations of the biology of Common Terns wintering in Trinidad. J. Field Ornithol.

[b2] Bønløkke J, Madsen JJ, Thorup K, Pedersen KT, Bjerrum M, Rahbek C (2006). Dansk Trækfugleatlas: the Danish bird migration atlas.

[b3] Boulet M, Gibbs HL, Hobson KA (2006). Integrated analysis of genetic, stable isotope, and banding data reveal migratory connectivity and flyways in the northern yellow warbler (Dendroica petechia; aestiva group). Ornithol. Monogr.

[b4] Brewer D, Diamond A, Woodsworth EJ, Collins BT, Dunn EH (2000). Canadian atlas of bird banding.

[b5] Bridge ES, Thorup K, Bowlin MS, Chilson PB, Diehl RH, Fléron RW (2011). Technology on the move: recent and forthcoming innovations for tracking migratory birds. Bioscience.

[b6] Brownie C, Hines JE, Nichols JD, Pollock KH, Hestbeck JB (1993). Capture-recapture studies for multiple strata including non-Markovian transitions. Biometrics.

[b7] Bugoni L, Vooren CM (2005). Distribution and abundance of six tern species in southern Brazil. Waterbirds.

[b8] Burnham K, Lebreton J-D, Northm PM (1993). A theory for combined analysis of ring recovery and recapture data. Marked individuals in the study of bird populations.

[b9] Cuthbert FJ, Wires LR, Poole A, Gill F (1999). Caspian tern (*Sterna caspia*. The birds of North America.

[b10] Diefenbach DR, Nichols JD, Hines JE (1988). Distribution patterns of American black duck and Mallard winter band recoveries. J. Wildl. Manag.

[b11] Douglas DC, Weinzierl R, Davidson SC, Kays R, Wikelski M, Bohrer G (2012). Moderating Argos location errors in animal tracking data. Methods Ecol. Evol.

[b12] Fraser KC, Stutchbury BJM, Silverio C, Kramer PM, Barrow J, Newstead D (2012). Continent-wide tracking to determine migratory connectivity and tropical habitat associations of a declining aerial insectivore. Proc. R. Soc. Lond. B Biol. Sci.

[b13] Fudickar AM, Wikelski M, Partecke J (2011). Tracking migratory songbirds: accuracy of light-level loggers (geolocators) in forest habitats. Methods Ecol. Evol.

[b14] Gauthier G, Lebreton JD (2008). Analysis of band-recovery data in a multistate capture-recapture framework. Can. J. Stat.

[b15] Gimenez O, Rossi V, Choquet R, Dehais C, Doris B, Varella H (2007). State-space modelling of data on marked individuals. Ecol. Model.

[b16] Gochfeld M, Burger J, Nisbet ICT, Poole A, Gill F (1998). Roseate tern (*Sterna dougallii*. The Birds of North America.

[b17] Gratto-Trevor C, Amirault-Langlais D, Catlin D, Cuthbert F, Fraser J, Maddock S (2012). Connectivity in piping plovers: do breeding populations have distinct winter distributions?. J. Wildl. Manag.

[b18] Hays H, DiCostanzo J, Cormons G, Antas PdeTZ, do Nascimento JLX, do Nascimento IdeLS (1997). Recoveries of roseate and common terns in South America. J. Field Ornithol.

[b19] Hays H, Lima P, Monteiro L, DiCostanzo J, Cormons G, Nisbet IC (1999). A nonbreeding concentration of roseate and common terns in Bahia, Brazil. J. Field Ornithol.

[b20] Henkel JR, Sigel BJ, Taylor CM (2012). Large-scale impacts of the deepwater horizon oil spill: can local disturbance affect distant ecosystems through migratory shorebirds?. Bioscience.

[b21] Hestbeck JB, Nichols JD, Malecki RA (1991). Estimates of movement and site fidelity using mark-resight data of wintering Canada geese. Ecology.

[b22] Hobson KA, Berthold P, Gwinner P, Sonnenschein E (2003). Making migratory connections with stable isotopes. Avian Migration.

[b23] Kendall WL, Conn PB, Hines JE (2006). Combining multistate capture-recapture data with tag recoveries to estimate demographic parameters. Ecology.

[b24] Korner-Nievergelt F, Hofer J (2009). Measuring within-winter movement rates of Tufted Duck *Aythya fuligula* and Common Pochard *A. ferina* based on ring re-encounter data. Wildfowl.

[b25] Korner-Nievergelt F, Sauter A, Atkinson PW, Guélat J, Kania W, Kéry M (2010a). Improving the analysis of movement data from marked individuals through explicit estimation of observer heterogeneity. J. Avian Biol.

[b26] Korner-Nievergelt F, Schaub M, Thorup K, Vock M, Kania W (2010b). Estimation of bird distribution based on ringre-encounters: precision and bias of the division coefficient and its relation to multi-state models. Bird Study.

[b27] Korner-Nievergelt F, Liechti F, Hahn S (2012). Migratory connectivity derived from sparse ring reencounter data with unknown numbers of ringed birds. J. Ornithol.

[b28] Laake J, Rexstad E (2008). RMark—an alternative approach to building linear models in MARK.

[b29] Lisovski S, Hewson CM, Klaassen RH, Korner-Nievergelt F, Kristensen MW, Hahn S (2012). Geolocation by light: accuracy and precision affected by environmental factors. Methods Ecol. Evol.

[b30] Macdonald CA, Fraser KC, Gilchrist HG, Kyser TK, Fox JW, Love OP (2012). Strong Migratory Connectivity in a Declining Arctic Passerine. Anim. Migr.

[b31] Marra PP, Francis CM, Mulvihill RS, Moore FR (2005). The influence of climate on the timing and rate of spring bird migration. Oecologia.

[b32] Marra PP, Studds CE, Webster M, Breed MD, Moore J (2010). Migratory connectivity. Encyclopedia of animal behavior.

[b33] McKellar AE, Marra PP, Hannon SJ, Studds CE, Ratcliffe LM (2012). Winter rainfall predicts phenology in widely separated populations of a migrant songbird. Oecologia.

[b34] Morris RD, Weseloh DV, Cuthbert FJ, Pekarik C, Wires LR, Harper L (2010). Distribution and abundance of nesting common and Caspian terns on the North American Great Lakes, 1976 to 1999. J. Great Lakes Res.

[b35] Nichols JD, Rhodes OE, Chesser RK, Smith MH (1996). Sources of variation in migratory movements of animal populations: statistical inference and a selective review of empirical results for birds. Population dynamics in ecological space and time.

[b36] Nichols JD, Reynolds RE, Blohm RJ, Trost RE, Hines JE, Bladen JP (1995). Geographic variation in band reporting rates for mallards based on reward banding. J. Wildl. Manag.

[b37] Nisbet IC (1984). Migration and winter quarters of North American Roseate Terns as shown by banding recoveries. J. Field Ornithol.

[b38] Nisbet IC, Poole A, Gill F (2002). Common tern (*Sterna hirundo*. The birds of North America.

[b39] Nisbet IC, Spendelow JA (1999). Contribution of research to management and recovery of the Roseate Tern: review of a twelve-year project. Waterbirds.

[b40] Nisbet IC, Mostello CS, Veit RR, Fox JW, Afanasyev V (2011). Migrations and winter quarters of five Common Terns tracked using geolocators. Waterbirds.

[b41] Norris DR, Marra PP, Kyser TK, Sherry TW, Ratcliffe LM (2004). Tropical winter habitat limits reproductive success on the temperate breeding grounds in a migratory bird. Proc. R. Soc. Lond. B Biol. Sci.

[b42] Olmos F (2002). Non-breeding seabirds in Brazil: a review of band recoveries. Ararajuba.

[b43] R Development Core Team (2012). R: a language and environment for statistical computing.

[b44] Royle JA, Dubovsky JA (2001). Modeling spatial variation in waterfowl band-recovery data. J. Wildl. Manag.

[b45] Rundel CW, Wunder MB, Alvarado AH, Ruegg KC, Harrigan R, Schuh A (2013). Novel statistical methods for integrating genetic and stable isotope data to infer individual-level migratory connectivity. Mol. Ecol.

[b46] Runge MC, Marra PP, Greenberg R, Marra PP (2005). Modeling seasonal interactions in the population dynamics of migratory birds. Birds of two worlds: the ecology and evolution of migration.

[b47] Rushing CS, Ryder TB, Saracco JF, Marra PP (2014). Assessing migratory connectivity for a long-distance migratory bird using multiple intrinsic markers. Ecol. Appl.

[b48] Ryder TB, Fox JW, Marra PP (2011). Estimating migratory connectivity of Gray Catbirds (*Dumetella carolinensis*) using geolocator and mark–recapture data. Auk.

[b49] Sharrock JTR (2010). The atlas of breeding birds in Britain and Ireland.

[b50] Sheehy J, Taylor CM, McCann KS, Norris DR (2010). Optimal conservation planning for migratory animals: integrating demographic information across seasons. Conserv. Lett.

[b51] Spendelow JA, Nichols JD, Nisbet IC, Hays H, Cormons GD (1995). Estimating annual survival and movement rates of adults within a metapopulation of Roseate Terns. Ecology.

[b52] Thorup K, Conn PB, Thomson DL, Cooch EG, Conroy MJ (2009). Estimating the seasonal distribution of migrant bird species: can standard ringing data be used?. Modeling demographic processes in marked populations.

[b53] Tøttrup AP, Thorup K, Rainio K, Yosef R, Lehikoinen E, Rahbek C (2008). Avian migrants adjust migration in response to environmental conditions en route. Biol. Lett.

[b54] Tøttrup AP, Klaassen RHG, Kristensen MW, Strandberg R, Vardanis Y, Lindstrom A (2012). Drought in Africa caused delayed arrival of European songbirds. Science.

[b55] Veen T (2013). Unravelling migratory connections: the next level. Mol. Ecol.

[b56] Visser ME, Perdeck AC, Van Balen JH, Both C (2009). Climate change leads to decreasing bird migration distances. Glob. Change Biol.

[b57] Webster MS, Marra PP, Greenberg R, Marra PP (2005). The importance of understanding migratory connectivity and seasonal interactions. Birds of two worlds: the ecology and evolution of migration.

[b58] White GC, Burnham KP (1999). Program MARK: survival estimation from populations of marked animals. Bird Study.

[b59] White GC, Kendall WL, Barker RJ (2006). Multistate survival models and their extensions in Program MARK. J. Wildl. Manag.

[b60] Wikelski M, Kays RW, Kasdin NJ, Thorup K, Smith JA, Swenson GW (2007). Going wild: what a global small-animal tracking system could do for experimental biologists. J. Exp. Biol.

[b61] Williams BK, Nichols JD, Conroy MJ (2002). Aanalysis and management of animal populations: modeling, estimation and decision making.

[b62] Wilson S, LaDeau SL, Tøttrup AP, Marra PP (2011). Range-wide effects of breeding-and nonbreeding-season climate on the abundance of a Neotropical migrant songbird. Ecology.

[b63] Wires LR, Cuthbert FJ (2000). Trends in Caspian tern numbers and distribution in North America: a review. Waterbirds.

