# Supplementary material for: Estimating migratory connectivity of birds when re-encounter probabilities are heterogeneous
Source: Ecol Evol. 2014 Apr 8;4(9):1659–70. doi: 10.1002/ece3.1059 (PMC4063466; doi:10.1002/ece3.1059)
Supplement: Supplementary file 1 [file ece30004-1659-SD1.doc]

**Supporting Information**

Estimating migratory connectivity of birds when re-encounter probabilities are heterogeneous

E.B. COHEN, J. A. HOSTETLER, J. A. ROYLE, P.P MARRA

**Table S1.** The number of birds banded in each breeding region and re-encountered in each non-breeding region. The percent of birds banded in each breeding region that were re-encountered in non-breeding regions. Of those re-encountered, the number recaptured, recovered, or unbanded after the encounter.

Common Tern Roseate Tern Caspian Tern

Eastern Central Western total Eastern Central Western Total

Banded 897364 130723 31270 1059357 104204 54971 20609 75580

E. South America 780 11 0 791 215 3 0 3

MX. and Central Am. 6 36 16 58 0 10 27 37

W. South Am. 11 29 6 46 2 14 0 14

U.S. and Caribbean 22 20 7 49 2 69 0 69

Total 819 96 29 944 219 96 27 123

Percentage 0.09% 0.07% 0.09% 0.09% 0.21% 0.17% 0.13% 0.16%

Total recaptured 390 8 2 400 158 10 2 12

Total recovered 323 65 21 409 43 81 22 103

Total unbanded 106 23 6 135 18 5 3 8
