# Supplementary material for: Estimating migratory connectivity of birds when re-encounter probabilities are heterogeneous
Source: Ecol Evol. 2014 Apr 8;4(9):1659–70. doi: 10.1002/ece3.1059 (PMC4063466; doi:10.1002/ece3.1059)
Supplement: Supplementary file 2 [file ece30004-1659-SD2.doc]

**Supporting Information**

Estimating migratory connectivity of birds when re-encounter probabilities are heterogeneous

E.B. COHEN, J. A. HOSTETLER, J. A. ROYLE, P.P MARRA

**Table S2.** Model precision and bias from 100 replicates of simulated data, where birds from each of four breeding areas (A-D) migrate to each of four stationary non-breeding areas (1-4). Scenarios varied in the number of birds banded in each of four breeding areas (10, 100, and 500 thousand), the strength of migratory connectivity (weak all π_ij_ = 0.25, moderate π_ij_ = 0.10, 0.15, 0.20, 0.55, and strong π_ij_ = 0.05, 0.05, 0.15, 0.75), and re-encounter probabilities. In each scenario, re-encounter probability is higher in non-breeding area 1 (very low: 0.0015, low: 0.01, or moderate: 0.08, respectively) and the same in non-breeding areas 2-4 (very low: 0.0002, low: 0.002, or moderate: 0.01, respectively). Annual survival was held constant at φ= 0.65. Mean number of re-encounters, coverage, RMSE and Bias of migratory connectivity estimates are shown. To assess bias between and within scenarios we calculated it separately for migratory connectivity parameters to non-breeding areas with lower (π _i2-4_) and higher re-encounter probabilities (π_i1_). Bias measures of greater than ± 0.01 are in bold.

| N | Re-encounter probability | Migratory Connectivity | Mean re-encounters | Coverage | RMSE | Bias (π_i1_) | Bias (π_i2-4_) |
| --- | --- | --- | --- | --- | --- | --- | --- |
| 10 | very low | weak | 68 | 80.50 | 0.27 | **0.16** | **-0.05** |
|  |  | moderate | 68 | 77.50 | 0.20 | 0.01 | 0.01 |
|  |  | strong | 69 | 63.50 | 0.16 | 0.00 | 0.00 |
| 10 | low | weak | 495 | 89.50 | 0.14 | **0.06** | **-0.02** |
|  |  | moderate | 495 | 94.50 | 0.07 | 0.00 | 0.00 |
|  |  | strong | 498 | 91.92 | 0.05 | 0.00 | 0.00 |
| 10 | moderate | weak | 3215 | 93.50 | 0.03 | -0.01 | 0.00 |
|  |  | moderate | 3206 | 94.42 | 0.02 | 0.00 | 0.00 |
|  |  | strong | 3221 | 96.42 | 0.02 | 0.00 | 0.00 |
| 100 | very low | weak | 688 | 87.25 | 0.20 | **0.14** | **-0.05** |
|  |  | moderate | 690 | 95.33 | 0.06 | 0.00 | 0.00 |
|  |  | strong | 681 | 94.33 | 0.05 | 0.00 | 0.00 |
| 100 | low | weak | 4964 | 94.42 | 0.03 | 0.00 | 0.00 |
|  |  | moderate | 4976 | 94.67 | 0.02 | 0.00 | 0.00 |
|  |  | strong | 4974 | 94.75 | 0.02 | 0.00 | 0.00 |
| 100 | moderate | weak | 32112 | 78.75 | 0.01 | -0.01 | 0.00 |
|  |  | moderate | 32104 | 89.50 | 0.01 | 0.00 | 0.00 |
|  |  | strong | 32139 | 89.25 | 0.01 | 0.00 | 0.00 |
| 500 | very low | weak | 3431 | 88.17 | 0.14 | **0.09** | **-0.03** |
|  |  | moderate | 3439 | 97.25 | 0.03 | 0.00 | 0.00 |
|  |  | strong | 3434 | 94.33 | 0.02 | 0.00 | 0.00 |
| 500 | low | weak | 24857 | 90.08 | 0.02 | -0.01 | 0.00 |
|  |  | moderate | 24853 | 94.67 | 0.01 | 0.00 | 0.00 |
|  |  | strong | 24832 | 94.83 | 0.01 | 0.00 | 0.00 |
| 500 | moderate | weak | 160487 | 56.50 | 0.01 | -0.01 | 0.00 |
|  |  | moderate | 160575 | 80.50 | 0.01 | 0.00 | 0.00 |
|  |  | strong | 160539 | 72.08 | 0.01 | 0.00 | 0.00 |
