# Supplementary material for: Estimating migratory connectivity of birds when re-encounter probabilities are heterogeneous
Source: Ecol Evol. 2014 Apr 8;4(9):1659–70. doi: 10.1002/ece3.1059 (PMC4063466; doi:10.1002/ece3.1059)
Supplement: Supplementary file 3 [file ece30004-1659-SD3.doc]

**Supporting Information**

Estimating migratory connectivity of birds when re-encounter probabilities are heterogeneous

E.B. COHEN, J. A. HOSTETLER, J. A. ROYLE, P.P MARRA

**Data S3.** R Code for MSLiveDead Mark models and Simulation analyses in this paper.

###############################################################################

### Migratory Connectivity MSLiveDead Mark models###

### Model Simulation###

# Import capture histories

TERNS <- read.csv ("CH.csv", as.is=TRUE)

# Capture history in string format for each individual (band number is the id)

# For all birds banded in regions A-D

# Re-encountered in regions 4-7 or never re-encountered or

# CH examples:

# id ch freq species

# 1 66540133 C000000000000000000700 1 CATE

# 2 51579257 C006000000000000000000 1 CATE

# ...

# 123 80512144 C070000000000000000000 1 CATE

# 124 None C000000000000000000000 55158 CATE

# Load RMark package

library(RMark)

# Set up model type, lengths of intervals between capture occasions (first

# interval is half year between summer banding and potential winter encounter),

# and species groups.

connect.proc<-process.data(TERNS,model="MSLiveDead", time.intervals=c(0.5,rep(1,10)), nocc=11, groups=c("species"))

# Create design dataframes for MARK model specification

# Specifying that p, r, and Psi are not affected by age (but S can be)

# Also specifying which psis are not estimated, but instead calculated by subtraction

connect.ddl<-make.design.data(connect.proc,parameters=list(

S=list(),

p=list(pim.type='constant'),

Psi=list(pim.type='constant', subtract.stratum=c("4","5","6","7","4","4","4","4")),

r=list(pim.type='constant')))

# Constrain psi to eliminate impossible transitions

connect.ddl$Psi<-connect.ddl$Psi[!(connect.ddl$Psi$tostratum=="A"),]

connect.ddl$Psi<-connect.ddl$Psi[!(connect.ddl$Psi$tostratum=="B"),]

connect.ddl$Psi<-connect.ddl$Psi[!(connect.ddl$Psi$tostratum=="C"),]

connect.ddl$Psi<-connect.ddl$Psi[!(connect.ddl$Psi$stratum=="4"),]

connect.ddl$Psi<-connect.ddl$Psi[!(connect.ddl$Psi$stratum=="5"),]

connect.ddl$Psi<-connect.ddl$Psi[!(connect.ddl$Psi$stratum=="6"),]

connect.ddl$Psi<-connect.ddl$Psi[!(connect.ddl$Psi$stratum=="7"),]

connect.ddl$Psi<-connect.ddl$Psi[!(connect.ddl$Psi$tostratum=="5" & connect.ddl$Psi$species=="ROTE"),]

connect.ddl$Psi<-connect.ddl$Psi[!(connect.ddl$Psi$stratum=="B" & connect.ddl$Psi$species=="ROTE"),]

connect.ddl$Psi<-connect.ddl$Psi[!(connect.ddl$Psi$stratum=="C" & connect.ddl$Psi$species=="ROTE"),]

connect.ddl$Psi<-connect.ddl$Psi[!(connect.ddl$Psi$stratum=="A" & connect.ddl$Psi$species=="CATE"),]

# Fully eliminate levels of factors where we've deleted the rows of the design dataframe

connect.ddl$Psi$tostratum <- factor(connect.ddl$Psi$tostratum)

connect.ddl$Psi$stratum <- factor(connect.ddl$Psi$stratum)

# Binary indicators for species (psi parameter)

connect.ddl$Psi$COTE <- ifelse(connect.ddl$Psi$species == 'COTE', 1, 0)

connect.ddl$Psi$ROTE <- ifelse(connect.ddl$Psi$species == 'ROTE', 1, 0)

connect.ddl$Psi$CATE <- ifelse(connect.ddl$Psi$species == 'CATE', 1, 0)

connect.ddl$Psi

# Define stratumCATE, which combines strata A & B for Caspian Tern

connect.ddl$Psi$stratumCATE <- connect.ddl$Psi$stratum

connect.ddl$Psi$stratumCATE[connect.ddl$Psi$stratumCATE=='A'] <- 'B'

connect.ddl$Psi$stratumCATE <- factor(connect.ddl$Psi$stratumCATE)

# Constrain recapture and recovery probabilty for breeding sites to 0

Br.p.Zeros<-as.numeric(row.names(connect.ddl$p[connect.ddl$p$stratum %in% c("A","B", "C") |

connect.ddl$p$stratum %in% c("5") & connect.ddl$p$species=='ROTE', ]))

p.fixed<-list(index=Br.p.Zeros,value=0)

Br.r.Zeros<-as.numeric(row.names(connect.ddl$r[connect.ddl$r$stratum %in% c("A","B", "C") |

connect.ddl$r$stratum %in% c("5") & connect.ddl$r$species=='ROTE',]))

r.fixed<-list(index=Br.r.Zeros,value=0)

# p effort covariate

strata.effort <- data.frame(stratum=c(4:7), effort=c(0.353307,4.510349,0.9792214,28.07768))

strata.effort$standard <-(strata.effort$effort-mean(strata.effort$effort))/sd(strata.effort$effort)

connect.ddl$p$effort <- strata.effort$standard[strata.effort$stratum==4]

connect.ddl$p$effort[connect.ddl$p$stratum=="5"] <- strata.effort$standard[strata.effort$stratum==5]

connect.ddl$p$effort[connect.ddl$p$stratum=="6"] <- strata.effort$standard[strata.effort$stratum==6]

connect.ddl$p$effort[connect.ddl$p$stratum=="7"] <- strata.effort$standard[strata.effort$stratum==7]

# r effort covariate

strata.effort <- data.frame(stratum=c(4:7), effort=c(0.8573582, 26.96942, 3.415333, 27.52401))

strata.effort$standard <-(strata.effort$effort-mean(strata.effort$effort))/sd(strata.effort$effort)

connect.ddl$r$effort <- strata.effort$standard[strata.effort$stratum==4]

connect.ddl$r$effort[connect.ddl$r$stratum=="5"] <- strata.effort$standard[strata.effort$stratum==5]

connect.ddl$r$effort[connect.ddl$r$stratum=="6"] <- strata.effort$standard[strata.effort$stratum==6]

connect.ddl$r$effort[connect.ddl$r$stratum=="7"] <- strata.effort$standard[strata.effort$stratum==7]

# Define models

S.dot<- list(formula=~1)

S.species<- list(formula=~species)

p.ewsite<-list(formula=~effort, fixed=p.fixed)

r.ewsite<-list(formula=~effort, fixed=r.fixed)

p.wsite<-list(formula=~stratum, fixed=p.fixed)

r.wsite<-list(formula=~stratum, fixed=r.fixed)

Psi.BWS<-list(formula=~-1+COTE:(stratum*tostratum)+ CATE:(stratumCATE*tostratum) + ROTE:tostratum)

# Run models of interest

# Species specific survival with effort covariate

inits<- as.vector(c(0.67,0,0.62,-8,0,rep(0,17),-8,0))

model<- make.mark.model(connect.proc,connect.ddl, parameters = list(S=S.species, p=p.ewsite, r=r.ewsite, Psi=Psi.BWS), initial=inits) #

modelPRSs<- run.mark.model(model, invisible = TRUE, realvcv=T)

modelPRSs

# Species specific survival without effort covariate (with strata covariate instead)

inits2<- as.vector(c(0.67,-0.17,0.62,-8,rep(-2,6),rep(0,17),-8,rep(0.5,6)))

model2<- make.mark.model(connect.proc,connect.ddl, parameters = list(S=S.species, p=p.wsite, r=r.wsite, Psi=Psi.BWS), initial=inits2) #

modelSs<- run.mark.model(model2, invisible = TRUE, realvcv=T)

modelSs

############################################################################

###Simulation###

# Generation of simulated data

# Simulate data with known migratory connectivity from 4 breeding to 4 non-breeding areas

# p = r

# S = 0.65

# Breeding A:D

# Wintering 1:4

# 100 simulations for each of 27 scenario, 3 connectivity x 3 number babded x 3 encounter probabilities

# PSI scenario(A,B,C), Variation in enc prob (1:3), Number banded per breeding area

####Number of birds banded in each of 4 breeding areas####

# Total number banded (in each breeding area)

#1. 40,000 (4x10,000) #e.g. AMRE and REVI just under 300,000, ACFL 30,000

#2. 400,000 (4x100,000)

#3. 2,000,000 (4x500,000)

####Re-encounter probabilities, varied by intercept###

#1. intercept: -8 values: 0.000203427 0.000203427 0.000203427 0.001501182

#2. intercept:-6 values: 0.001501182 0.001501182 0.001501182 0.010986943

#3. intercept:-4 values: 0.01098694 0.01098694 0.01098694 0.07585818

####Degree of migratory connectivity####

#A. When true connectivity weak, equal to each winter area: 0.25

#B. When true connectivity is moderate, less mixing between winter areas: 0.10, 0.15, 0.20, 0.55

#C. When true connectivity is strong, little mixing between winter areas: 0.05, 0.05, 0.15, 0.75

# Define function to simulate multistate capture-recapture data

simul.ms2 <- function(PSI.STATE, PHI.STATE, P.OBS, R.OBS, marked, breed.sites="ABCD", unobservable = NA){

# Unobservable: number of state that is unobservable

n.occasions <- dim(PHI.STATE)[3] + 1

n.breed <- nchar(breed.sites)

notseen <- dim(P.OBS)[2]

CH.P <- CH.R <- CH.TRUE <- matrix(NA, ncol = n.occasions, nrow = sum(marked))

first <- 1 # Hopefully speeding things up by assuming all animals marked first occasion

for (i in 1:sum(marked)){

s <- min(which(i <= cumsum(marked[first,]))) # Breeding site

CH.P[i,first] <- s + notseen

CH.R[i,first] <- 0

# Assign each individual to a wintering location

w.site <- which(rmultinom(1, 1, PSI.STATE[s,])==1)

CH.TRUE[i,first] <- w.site

for (t in (first+1):n.occasions){

# Multinomial trials for state transitions

state <- which(rmultinom(1, 1, PHI.STATE[CH.TRUE[i,t-1],,t-1])==1)

CH.TRUE[i,t] <- state

# Multinomial trials for capture process

event <- which(rmultinom(1, 1, P.OBS[CH.TRUE[i,t],,t-1])==1)

CH.P[i,t] <- event

# Multinomial trials for recovery process

event <- which(rmultinom(1, 1, R.OBS[CH.TRUE[i,t],,t-1])==1)

CH.R[i,t] <- event

} #t

} #i

# Interlace the recapture and recovery histories

CH <- matrix(NA, ncol = n.occasions*2, nrow = sum(marked))

CH[,seq(1, n.occasions*2, 2)] <- CH.P

CH[,seq(2, n.occasions*2, 2)] <- CH.R

# Replace the NA and the highest state number (dead) in the file by 0

CH[is.na(CH)] <- 0

CH[CH==notseen] <- 0 #change the not seen to 0 because they were 5

# Convert everything to character vectors

cmr = vector(mode="character",length=sum(marked))

for (i in 1:sum(marked)) cmr[i]=paste(CH[i,],collapse="")

cmr <- chartr(paste(1:n.breed + notseen, collapse=""), breed.sites, cmr)

return(list(CH=CH, CH.CHAR=cmr, CH.TRUE=CH.TRUE))

}

# Define PSI scenario values

psiA1 <- 0.25

psiA2 <- 0.25

psiA3 <- 0.25

psiA4 <- 0.25

psiB1 <- 0.25

psiB2 <- 0.25

psiB3 <- 0.25

psiB4 <- 0.25

psiC1 <- 0.25

psiC2 <- 0.25

psiC3 <- 0.25

psiC4 <- 0.25

psiD1 <- 0.25

psiD2 <- 0.25

psiD3 <- 0.25

psiD4 <- 0.25

# Define define re-encounter probability scenario values####

r.effort <- data.frame(stratum=2:5, effort=c(300, 300, 300, 9500))

r.effort$standard =(r.effort$effort-mean(r.effort$effort))/sd(r.effort$effort)

r.intercept <- -8

r.slope <- 1

r <- plogis(r.intercept + r.slope * r.effort$standard)

r

r1 <- r[4]

r2 <- r[1]

r3 <- r[2]

r4 <- r[3]

p.effort <- data.frame(stratum=2:5, effort=c(300, 300, 300, 9500))

p.effort$standard =(p.effort$effort-mean(p.effort$effort))/sd(p.effort$effort)

p.intercept <- -8

p.slope <- 1

p <- plogis(p.intercept + p.slope * p.effort$standard)

p

p1 <- p[4]

p2 <- p[1]

p3 <- p[1]

p4 <- p[1]

# Define mean survival, transitions, recapture, as well as number of occasions,states and observations

n.sims <- 100

phi <- 0.65 #survival

# First period is Breeding, then 10 winter encounters

n.occasions <- 11

n.states <- 4 #there are 4 wintering areas

n.breed <- 4 #there are 4 breeding areas

n.obs <- 5 #there are four p and not seen, four r and not recovered

# Define define number banded scenario values####

n.band <- matrix(rep(c(10000, 100000, 500000), each=n.breed), nrow=n.breed)

n.band

# Define matrices with survival, transition and recapture probabilities

# These are 4-dimensional matrices, with

# Dimension 1: state of assignment (psi)

# Dimension 2: state of survival (phi)

# Dimension 3: observation matrix 1 (recapture, p)

# Dimension 4: observation matrix 2 (recovery, r)

# Dimension 5: time

totrel <- sum(marked) #released once

PSI.STATE <- matrix(c(

psiA1, psiA2, psiA3, 1-(psiA1+psiA2+psiA3),

psiB1, psiB2, psiB3, 1-(psiB1+psiB2+psiB3),

psiC1, psiC2, psiC3, 1-(psiC1+psiC2+psiC3),

psiD1, psiD2, psiD3, 1-(psiD1+psiD2+psiD3)), nrow = n.breed, byrow = TRUE)

# 2. State process matrix: survival, phi

PHI.STATE <- array(NA, dim=c(2*n.states+1, 2*n.states+1, n.occasions-1))

PHI.STATE[,,1] <- matrix(c(

sqrt(phi), 0, 0, 0, 1-sqrt(phi), 0, 0, 0, 0,

0, sqrt(phi), 0, 0, 0,1-sqrt(phi), 0, 0, 0,

0, 0, sqrt(phi), 0, 0,0,1-sqrt(phi), 0, 0,

0, 0, 0, sqrt(phi), 0,0,0,1-sqrt(phi), 0,

0, 0, 0, 0, 0, 0, 0, 0, 1,

0, 0, 0, 0, 0, 0, 0, 0, 1,

0, 0, 0, 0, 0, 0, 0, 0, 1,

0, 0, 0, 0, 0, 0, 0, 0, 1,

0, 0, 0, 0, 0, 0, 0, 0, 1), nrow = 2*n.states+1, byrow = TRUE)

for (t in 2:(n.occasions-1)){

PHI.STATE[,,t] <- matrix(c(

phi, 0, 0, 0, 1-phi, 0, 0, 0, 0,

0, phi, 0, 0, 0,1-phi, 0, 0, 0,

0, 0, phi, 0, 0,0,1-phi, 0, 0,

0, 0, 0, phi, 0,0,0,1-phi, 0,

0, 0, 0, 0, 0, 0, 0, 0, 1,

0, 0, 0, 0, 0, 0, 0, 0, 1,

0, 0, 0, 0, 0, 0, 0, 0, 1,

0, 0, 0, 0, 0, 0, 0, 0, 1,

0, 0, 0, 0, 0, 0, 0, 0, 1), nrow = 2*n.states+1, byrow = TRUE)

} #t

#3. Observation process matrix, (recapture, p)

P.OBS <- array(NA, dim=c(2*n.states+1, n.obs, n.occasions-1))

for (t in 1:(n.occasions-1)){

P.OBS[,,t] <- matrix(c(

p1, 0, 0, 0, 1-p1,

0, p2, 0, 0, 1-p2,

0, 0, p3, 0, 1-p3,

0, 0, 0, p4, 1-p4,

0, 0, 0, 0, 1,

0, 0, 0, 0, 1,

0, 0, 0, 0, 1,

0, 0, 0, 0, 1,

0, 0, 0, 0, 1), nrow = 2*n.states+1, byrow = TRUE)

} #t

#4.Observation process matrix, (recovery, r)

R.OBS <- array(NA, dim=c(2*n.states+1, n.obs, n.occasions-1))

for (t in 1:(n.occasions-1)){

R.OBS[,,t] <- matrix(c(

0, 0, 0, 0, 1,

0, 0, 0, 0, 1,

0, 0, 0, 0, 1,

0, 0, 0, 0, 1,

r1, 0, 0, 0, 1-r1,

0, r2, 0, 0, 1-r2,

0, 0, r3, 0, 1-r3,

0, 0, 0, r4, 1-r4,

0, 0, 0, 0, 1), nrow = 2*n.states+1, byrow = TRUE)

} #t

for (scenario in 1:ncol(n.band)) {

marked <- matrix(0, ncol = n.breed, nrow = n.occasions)

marked[1,] <- n.band[,scenario] # This is the number banded in each of the four breeding areas

# Names of each of the repititions

SIM2<-data.frame(Simulation=rep('', n.sims), Encounters2=NA, Encounters3=NA, Encounters4=NA,

Encounters5=NA, BandedA=NA, BandedB=NA, BandedC=NA, BandedD=NA, stringsAsFactors = FALSE)

for (e in 1:n.sims) {

# Execute simulation function #sim has CH, CH.TRUE, CH.CHAR

sim <- simul.ms2(PSI.STATE, PHI.STATE,P.OBS, R.OBS, marked)

SIM<-data.frame(ch = sim$CH.CHAR,stringsAsFactors = FALSE, row.names = NULL, freq=1)

# Change 1 to 5 because 1 is a problem for RMarked

SIM$ch <- gsub("1", "5", SIM$ch)

# Name and save file

simname <- paste("sim.psiA.enc1.band",marked[1,1]/1000,".",e,".csv",sep="")

SIM2$Simulation[e] <- simname

write.csv(SIM, simname, row.names= FALSE)

# Make another file with the number of encounters per winter area

# Add the name of the simulaiton

# and the number of encounters in each winter area

# and the number banded in each breeding area

SIM2$Encounters2[e]<-as.numeric(length(grep("2",SIM$ch)))

SIM2$Encounters3[e]<-as.numeric(length(grep("3",SIM$ch)))

SIM2$Encounters4[e]<-as.numeric(length(grep("4",SIM$ch)))

SIM2$Encounters5[e]<-as.numeric(length(grep("5",SIM$ch)))

SIM2$BandedA[e]<-length(grep("A",SIM$ch))

SIM2$BandedB[e]<-length(grep("B",SIM$ch))

SIM2$BandedC[e]<-length(grep("C",SIM$ch))

SIM2$BandedD[e]<-length(grep("D",SIM$ch))

# Close loop

}

uwrite.csv(SIM2,paste("sum.psiA.enc1.band",marked[1,1]/1000,".csv",sep=""), row.names= FALSE)

}

# Naming:PSI scenario(A,B,C), Variation in enc prob (1:3), Number banded per breeding area (in thousands) (10,100,500)

# Last digit is the trial number 1:100
